# The Influence of MicroRNAs on Mitochondrial Calcium

**DOI:** 10.3389/fphys.2018.01291

**Published:** 2018-09-21

**Authors:** Carolina Jaquenod De Giusti, Barbara Roman, Samarjit Das

**Affiliations:** ^1^Centro de Investigaciones Cardiovasculares CIC-CONICET, Facultad de Ciencias Médicas, Universidad Nacional de La Plata, La Plata, Argentina; ^2^Department of Pathology, Johns Hopkins University, Baltimore, MD, United States

**Keywords:** microRNA, miRNA, mitochondria, MitomiR, mitochondrial calcium, heart failure

## Abstract

Abnormal mitochondrial calcium ([Ca^2+^]_m_) handling and energy deficiency results in cellular dysfunction and cell death. Recent studies suggest that nuclear-encoded microRNAs (miRNA) are able to translocate in to the mitochondrial compartment, and modulate mitochondrial activities, including [Ca^2+^]_m_ uptake. Apart from this subset of miRNAs, there are several miRNAs that have been reported to target genes that play a role in maintaining [Ca^2+^]_m_ levels in the cytoplasm. It is imperative to validate miRNAs that alter [Ca^2+^]_m_ handling, and thereby alter cellular fate. The focus of this review is to highlight the mitochondrial miRNAs (MitomiRs), and other cytosolic miRNAs that target mRNAs which play an important role in [Ca^2+^]_m_ handling.

## Introduction

One of the cell's major sources of energy comes from mitochondria. However, the role of mitochondria is not limited only to ATP generation; mitochondria participate and control numerous metabolic pathways and signaling cascades, redox balance, β-oxidation of fatty acids, the synthesis of amino acids, heme and steroids, and cellular apoptosis. The ability of mitochondria to act as Ca^2+^ buffer has important consequences on the pattern of the [Ca^2+^]_cyto_ signals. In addition, mitochondria are also a major storage hub of cellular calcium (Ca^2+^), and Ca^2+^ homeostasis is fundamental for a wide range of cellular activities such as control of oxidative phosphorylation (OXPHOS), modulation of cytosolic Ca^2+^ ([Ca^2+^]_cyto_) signals, cell death, secretion, and the production of reactive oxygen species (ROS) (Mammucari et al., 2018). Approximately 50 years ago Engstrom et al., demonstrated that mitochondria were able to take up Ca^2+^(Deluca and Engstrom, 1961), which validated the observation made by Slater and Cleland as early as 1953. In this report, the authors evaluated the effect of Ca^2+^ on respiratory activity of heart sarcosomes (previous nomenclature of mitochondria) (Slater and Cleland, 1953). This work is the basis of future research in the area of Ca^2+^-mediated regulation of mitochondrial bioenergetics and mitochondrial calcium ([Ca^2+^]_m_) uptake. Additional studies have demonstrated a clear role for [Ca^2+^]_m_ as a pleiotropic signal that regulates many essential functions of cellular physiology. [Ca^2+^]_m_ concentration is about 10^−7^ M and it is the result of the dynamic equilibrium between two continuous and opposite processes: mitochondrial Ca^2+^ influx vs. efflux (Granatiero et al., 2017).

The functional relevance of [Ca^2+^]_m_ in energy processes has been shown in studies that reflect a positive effect on the tricarboxylic acid (TCA) cycle that increases the synthesis of reduced substrates (NADH and FADH2), enhances electron transport chain (ETC) activity, and subsequently increases H^+^ pumping (Rizzuto et al., 2012). Three mitochondrial matrix dehydrogenases are activated by Ca^2+^: α-ketoglutarate (α-KGDH) and isocitrate-dehydrogenases (ICDH) are regulated by direct binding of Ca^2+^, while pyruvate dehydrogenase (PDH) is regulated by a Ca^2+^-dependent phosphatase (PDP1) (Glancy and Balaban, 2012; Das et al., 2014). Stimulation of these dehydrogenases increases NADH and FADH2 availability, which is not only the central mechanism to provide electron carriers for ATP production, but also important for regenerating the antioxidative capacity of the mitochondrial matrix (Nickel et al., 2014). In addition, Ca^2+^ activates α-glycerolphosphate dehydrogenase, a component of the glycerol phosphate shuttle that supplies NAD+ for glycolysis (Wernette et al., 1981). Apart from these proteins, [Ca^2+^]_m_ can also modulate the adenine nucleotide transporter (Mildaziene et al., 1995) and the mitochondrial ATP synthase (complex V) expression (Das and Harris, 1990), harnessing the H^+^ gradient to upregulate ATP production.

It is generally understood that ROS production occurs through complexes I and III of the electron transport chain, and by NADPH-oxidase (NOX 4) (Ago et al., 2010). Additionally, ROS can also be produced by β-oxidation of fatty acids (Perevoshchikova et al., 2013). It has also been demonstrated that ROS production is dependent on the activity of α-glycerolphosphate dehydrogenase (Zorov et al., 2014). ROS production is directly related to increased [Ca^2+^]_m_ by the activation of metabolic fluxes through the TCA cycle, and increased oxygen consumption by the ETC (Hempel and Trebak, 2017). In fact, there is reciprocal regulation between ROS and [Ca^2+^]_m_ signaling, and calcium signaling is essential for ROS production through activation of mitochondrial dehydrogenases in the ETC (Görlach et al., 2015). On the other hand, under physiological conditions, a small amount of H_2_O_2_ release from mitochondria acts as a signal to stimulate physiological Ca^2+^ release from endoplasmisc reticulum (ER). This happens via the ryanodine receptors (RyR) which increases [Ca^2+^]_m_ uptake to increase ATP production to meet cellular energetic demands (Camara et al., 2010; Görlach et al., 2015).

Finally, [Ca^2+^]_m_ also stimulates cell death pathways, such as apoptosis and necrosis, via activating the intrinsic pathway. [Ca^2+^]_m_ overload or mitochondrial depolarization can open the mitochondrial permeability transition pore (mPTP) (Rasola and Bernardi, 2011). The mPTP is formed by interactions between IMM and OMM proteins such as VDAC1, ANT (adenine nucleotide translocase), and Cyp-D (cyclophilin D), however, deletion of each one of these proteins does not block the permeability increase caused by some inducers, indicating that the mPTP may not be formed by specific proteins, but just by the interaction between both membranes (Tomasello et al., 2009). While briefopening and closing of mPTP (more commonly known as “mitochondrial flickering”) can help to transient transport [Ca^2+^]_m_, complete mPTP openings can result in release of proapoptotic factors and [Ca^2+^]_m_ resulting in apoptosis (Bernardi et al., 2015). When the stress signal results in simultaneous mPTP opening from several mitochondria, ATP depletion would result in necrosis (Rasola and Bernardi, 2011). Therefore, it is quite evident that [Ca^2+^]_m_ plays an important role in both the necrotic and apoptotic cell death pathways. The role of [Ca^2+^]_m_ and ROS in apoptosis is particularly important considering that a small amount of cytochrome c released by mitochondria can bind and promote the opening of inositol triphosphate (IP3R) channels. Activated IP3R can release Ca^2+^ from the ER, which increases [Ca^2+^]_cyto_. Consequently, this cycle further leads to a massive release of cytochrome c, and activation of caspases and apoptosis (Boehning et al., 2003).

## Mitochondrial calcium transport

The mitochondrion is an organelle formed by two different phospholipid bilayers, an outer mitochondrial membrane (OMM) that allows the entrance of ions and small proteins (MW < 10 kDa) and an inner mitochondrial membrane (IMM), which is ion-impermeable (Mammucari et al., 2018). Calcium enters the mitochondria through voltage dependent anion channels (VDAC) in the OMM (Shoshan-Barmatz et al., 2018) and through the mitochondrial calcium uniporter (MCU) complex from the IMM using the mitochondrial membrane potential (ΔΨ_m_ around −150 to −180 mV) as the driving force generated by the electron transport chain (Liu et al., 2017). MCU has high Ca^2+^ affinity and selectivity, enabling high Ca^2+^ transport in spite of low [Ca^2+^]_cyto_ (Kirichok et al., 2004; Baughman et al., 2011; De Stefani et al., 2011). The MCU complex is formed by MCU core proteins and several regulatory proteins such as MCUb, EMRE, MICU1, MICU2, and MCUR1 (Mallilankaraman et al., 2012; Hajnóczky et al., 2014; Tomar et al., 2016). Calcium release is mediated by the Na^+^/Ca^2+^ exchanger (NCLX) (Palty et al., 2010) and through the H^+^/Ca^2+^ exchanger (HCX) in tissues where mitochondrial NCLX activity is low, such as the liver, kidney, lung, and smooth muscle (Takeuchi et al., 2015). HCX is also known as LETM1 (the leucine zipper-EF hand-containing transmembrane protein 1), but its role as an H^+^/Ca^2+^ exchanger or a K^+^/H^+^ exchanger is still controversial (Jiang et al., 2009; Austin et al., 2017). NCLX activity can be regulated by post-translational modifications, such as phosphorylation. Alterations in phosphorylation status in NCLX result in its decreased activity, increased [Ca^2+^]_m_ overload, partial mitochondrial depolarization and increased oxidative stress (Kostic et al., 2015).

## The role of the endoplasmic reticulum

The ER is the main Ca^2+^ storage location in the cell. There are overlapping regions between the ER and mitochondria where highly efficient Ca^2+^ transfer can occur (Patergnani et al., 2011). Various pathophysiological conditions increase the cross-talk between ER and mitochondria. The VDAC-IP3R interaction is important for [Ca^2+^]_m_ accumulation (Szabadkai et al., 2006). Increased sarcoplasmic reticulum (SR)/ER calcium leakage through RyR or IP3R channels has been implicated in mitochondrial calcium overload (Santulli et al., 2015; Ivanova et al., 2017). In fact, RyR or IP3R oxidation by mitochondrial ROS increases ER Ca^2+^ leakage and leads to higher [Ca^2+^]_m_ entry (Camara et al., 2010). Protein kinase A (PKA) (Banerjee and Ghosh, 2006; Gupta, 2017) and GSK-3β (Das et al., 2008) can regulate VDAC activity by regulating the phosphorylation status of VDAC. Similarly, another OMM translocator protein of 18 kDa size, TSPO, has been shown to alter [Ca^2+^]_m_ intake. TSPO is capable of complexing with PKA and Acyl-CoA binding domain containing 3 (ACBD3), recruiting them to the OMM, which leads to the phosphorylation of VDAC (Gatliff et al., 2017). Additionally, it has been shown that transient increases in VDAC expression result in increased [Ca^2+^]_m_ intake (Rapizzi et al., 2002).

## The role of the MCU complex

The MCU, a 40 kDa protein, has two transmembrane domains that need additional interactions to form a functional channel. It lacks a Ca^2+^ sensing domain (EF-hand domain), indicating that Ca^2+^ regulation capacity is not mediated by MCU (Mammucari et al., 2018). It has been shown that phosphorylation by CaMKII can modulate MCU activity (Joiner et al., 2012) as well as other proteins involved in the complex (Mammucari et al., 2018). MCU expression and activity may differ between tissues. The highest MCU mRNA expression levels are seen in skeletal muscle, followed by the liver, heart, spleen, lung, kidney, brain, and white fat (Raffaello et al., 2013). MCU activity measured by patch clamp in isolated mitochondria from mouse hearts, skeletal muscle, liver, kidney, and brown fat showed that heart mitochondria exhibited the lowest MCU current. However, this diminished current could be compensated for by the high numbers of mitochondria in cardiac muscle (Fieni et al., 2012).

MCUb is a 330 amino acid long protein that shares 50% similarity with MCU (Raffaello et al., 2013). By studying the MCU:MCUb interaction using FRET analysis and immunoprecipitation (IP), Raffaello et al., showed that co-expression of MCUb with MCU drastically diminishes MCU activity. (Raffaello et al., 2013).

EMRE (Essential MCU Regulator) is a 10 kDa protein with a mitochondrial target sequence at the 3'-end. Using *in vitro* models such as HEK293T and HeLa cells, the interaction between EMRE and MCU was identified. Silencing EMRE abolishes [Ca^2+^]_m_ uptake (Sancak et al., 2013). Furthermore, EMRE also facilitates the interaction between MICU1 and MICU2 with MCU. Cells that lack EMRE have no associations between MCU and MICU1 or MICU2, while the MCU:MCUb or MICU1:MICU2 interactions are not affected (Sancak et al., 2013). Unlike MICU1 or MICU2, EMRE expresses a different Ca^2+^ sensing domain directed towards the mitochondrial matrix. Therefore, EMRE modulates MCU activity via [Ca^2+^]_m_ levels (Sancak et al., 2013). In fact, EMRE mutation at the Ca^2+^ sensing acidic motif does not affect MCU complex formation, but results in increased [Ca^2+^]_m_ (Kameyama and Gemba, 1991). It is also important to note that the modulatory effect of EMRE also depends on the [Ca^2+^]_cyto_ binding affinity to the MICU1:MICU2 complex (Kameyama and Gemba, 1991). In summary, EMRE plays an important physical and modulatory role in the formation and activity of the MCU complex.

MICU1 has two Ca^2+^ sensing EF-hand motifs directed toward the intermembrane space (IMS). It has been demonstrated that MICU1 inhibits [Ca^2+^]_m_ entry at low resting [Ca^2+^]_cyto_ concentrations (within the range found in cells at rest), protecting mitochondria from [Ca^2+^]_m_ overload (Mallilankaraman et al., 2012; Csordás et al., 2013; Liu et al., 2016). On the other hand, MICU1 allows for [Ca^2+^]_m_ uptake when [Ca^2+^]_cyto_ concentrations increase to the micromolar range (Csordás et al., 2013). This would help mitochondria to rapidly respond to large [Ca^2+^]_cyto_ increases (like the one from systolic release). Interestingly, results from human skin fibroblasts with MICU1 mutations confirmed the gatekeeper role of MICU1 at low [Ca^2+^]_cyto_, but also showed increased [Ca^2+^]_m_ uptake at higher [Ca^2+^]_cyto_ (Logan et al., 2014). In contrast, MICU2 has been shown to form a complex with MCU in the presence of MICU1. MICU2 follows the same pattern of [Ca^2+^]_cyto_ threshold sensing as MICU1. MICU2 knock-out cells showed higher [Ca^2+^]_m_ uptake at lower [Ca^2+^]_cyto_ (Kamer and Mootha, 2014). Similarly, using planar lipid bilayers, Patron et al., concluded that MICU2 plays a gatekeeping role for MCU. MICU2 alters the ratio between MICU1:MICU2, which allows for the formation of MICU1-MICU1 dimers, which is responsible for the [Ca^2+^]_m_ uptake rate (Patron et al., 2014). Taken together, these MCU regulatory proteins regulate the rate of [Ca^2+^]_m_ entry in response to different [Ca^2+^]_cyto_ levels. The relative expression of these MCU regulatory proteins in different tissues, such as the heart, brain, and skeletal muscle, confers different mitochondrial calcium handling capabilities (Paillard et al., 2017).

## Mitochondrial calcium overload in the pathophysiology of disease states

[Ca^2+^]_m_ overload and diminished [Ca^2+^]_m_ have both been linked to the pathophysiology of different disease states. Particularly, high [Ca^2+^]_m_ load due to increased SR leakage through RyR channels has been related to ROS production, which ultimately leads to heart failure (Santulli et al., 2015). Human disease conditions, such as proximal myopathy, learning difficulties, and a progressive extrapyramidal movement disorder, have also been linked to [Ca^2+^]_m_ overload (Logan et al., 2014). Similarly, it has been validated that [Ca^2+^]_m_ overload is responsible for excessive ROS production and mitochondrial depolarization, which results in neuronal death (Saffari et al., 2017). This mechanism is one of the highlights of Parkinson disease progression (Kostic et al., 2015; Ammal Kaidery and Thomas, 2018; Ludtmann and Abramov, 2018).

## Influence of [Ca^2+^]_m_ in cancer

In order to meet the energy demand, cancer cells activate mitochondrial metabolism. This increased metabolism is achieved by activation of the TCA cycle dehydrogenases through increased [Ca^2+^]_m_ entry. Considering the up-regulation of both IP3R and MCU in several types of cancer cells, it seems that ER/mitochondria communication is key for increased mitochondrial metabolism and cancer progression (Bustos et al., 2017). VDAC1 expression is related to poor prognosis in breast, colon, and lung cancers, where it has been demonstrated that VDAC1 promotes cell growth by influencing energy metabolism and inhibiting apoptosis (Mazure, 2017; White, 2017). Additionally, pulmonary arterial cancer cells and colon cancer cells avoid [Ca^2+^]_m_ overload through downregulation of MCU, which confers resistance to cell death (Liu et al., 2017). Different oncogenes, such as *Ras* and *Akt*, have been shown to regulate apoptosis through modulation of [Ca^2+^]_m_ entry, and thus, inhibition of [Ca^2+^]_m_ overload (Rimessi et al., 2015). In the same manner, the known tumor suppressor gene, p53, is capable of interacting with sarco/endoplasmic reticulum Ca^2+^-ATPase (SERCA). p53 has been shown to influence the oxidative state of SERCA, and thus increase SERCA activity in the ER/mitochondria associated membranes (MAM) fraction. This is related to increased ER calcium release that results in [Ca^2+^]_m_ overload; a priming step for the release of caspase cofactors—and induction of apoptosis (Giorgi et al., 2015; Rimessi et al., 2015). Similarly, the tumor suppressor gene, Fhit, increases MCU affinity toward Ca^2+^ and induces apoptosis (Rimessi et al., 2009). Based on this, drugs that increase [Ca^2+^]_m_ entry through ER transfer and/or augmented Ca^2+^ influx can be used for cancer therapy (Rimessi et al., 2015). However, a strict balance in [Ca^2+^]_m_ homeostasis is involved in cancer as Ca^2+^ influx is also needed for cellular energy production and cancer progression (Bustos et al., 2017).

## Influence of [Ca^2+^]_m_ in cardiovascular health

Alterations in calcium handling and the processes regulated by Ca^2+^ have been documented in several cardiovascular diseases (Griffiths, 2009; Kim et al., 2014; Santulli et al., 2015). Santulli et al., showed increased [Ca^2+^]_m_ in a heart failure model by permanent occlusion of the proximal left anterior descending (LAD) coronary artery (Santulli et al., 2015). In fact, heart failure is characterized by abnormal [Ca^2+^]_m_ handling and poor energy production, which ultimately leads to contractile dysfunction and myocyte death. This increased [Ca^2+^]_m_ concentration was related to mitochondrial dysfunction seen as membrane depolarization, reduced ATP production, and ROS generation. In ischemia/reperfusion (IR) injury, both myocytes and whole hearts are characterized by increased [Ca^2+^]_m_ uptake resulting in excessive ROS production and mPTP opening, which ultimately leads to cell death (Ferrari, 1996; Griffiths, 2009; Kim et al., 2014).

## Introduction to mitochondrial microRNAs

Understanding some of the key cellular abnormalities in various disease conditions and developing specific therapies toward reversing these abnormalities can improve the disease state. microRNAs (miRNA) represent a large set of master regulators of gene expression, which can greatly influence cell death. The binding of miRNAs to their targeted mRNAs can result in translational repression and/or degradation of target genes. These miRNAs are predicted to regulate almost 30–40% of mammalian genes (Kim, 2005; Lewis et al., 2005).

With recent advancements in RNA detecting platforms, we now can detect miRNAs in the mitochondrial compartment of cells. The subset of miRNAs, which translocate into the mitochondria and are functionally active, i.e., RNA-induced silencing complex, or RISC, is inside the mitochondrial compartment, are termed mitochondrial miRNA or MitomiR. The mitochondrial miRNA translocation phenomenon has not been extensively studied (Macgregor-Das and Das, 2018), but MitomiRs can influence various mitochondrial functions, such as the TCA, ETC, lipid metabolism, and amino acid metabolism (Baradan et al., 2017). It has been demonstrated that MitomiRs can post-translationally regulate gene function inside the mitochondrial fraction either by targeting a nuclear-encoded gene present in the mitochondria or by targeting a mitochondrial encoded-gene (Das et al., 2012, 2014, 2017). Interestingly, it has been documented that overexpression of a MitomiR, miR-181c, in the heart can influence [Ca^2+^]_m_ handling, highlighting the role of miR-181c in heart failure (Das et al., 2014). Thus, in this article, we have focused on the miRNAs that have been found in the mitochondrial compartment, and target either mitochondrial or nuclear genes, which ultimately alters [Ca^2+^]_m_ handling and cellular fate in different disease conditions.

## The regulation of the MCU complex proteins by MicroRNAs

Bargaje et al., showed a role for miR-34a and miR-29a in apoptosis by targeting the 3′-UTR of VDAC1 mRNA in HEK-293 cells. These miRNAs are dowregulated in hepatocellular carcinoma and ovarian cancer. However, [Ca^2+^]_m_ levels were not documented in this study (Bargaje et al., 2012). Furthermore, miR-7 has been shown to reduce VDAC1 expression in SH-SY5Y neuroblastoma cells and mouse primary cortical neurons. This diminishes ${\text{Ca}}_{\text{m}}^{2+}$ efflux, and reduces ROS production, membrane depolarization, cytochrome c release, and apoptosis, all of which are factors involved in the development of Parkinson's disease (PD) (Chaudhuri et al., 2016). Due to its effects on [Ca^2+^]_m_, miR-7 could potentially be used as a therapeutic agent in the treatment of Parkinson's disease. Importantly, miR-7 expression has never been measured in PD's patients.

It was proposed from *in silico* analysis that five miRNAs, miR-15, miR-17, miR-21, miR-25, and miR-137, could target MCU and/or MICU1 in tumor cells (Marchi et al., 2013). However, only miR-25 was able to reduce [Ca^2+^]_m_ levels in HeLa cells through MCU downregulation (Marchi et al., 2013). In both human colon cancer and prostate cancer, the up-regulation of miR-25 and down-regulation of MCU protein have been observed. Interestingly, the authors performed biochemical assays to demonstrate that miR-25 can directly bind to the 3' UTR of MCU mRNA (Marchi et al., 2013). Treating with antagomiR-25 can increase [Ca^2+^]_m_ entry and apoptosis (Marchi et al., 2013). In contrast, in breast cancer the downregulation of miR-340 is correlated with increased MCU expression. Targeting MCU, miR-340 increases the rate of glycolysis, a metabolic shift known as the Warburg effect, which promotes cell migration and invasion (Yu et al., 2017). Pulmonary arterial hypertension (PAH) is characterized by excessive pulmonary artery smooth muscle cell proliferation, migration, and apoptosis resistance, resulting in a cancer-like phenotype (Hong et al., 2017). In this model, Hong et al., showed decreased expression of MCU which resulted in elevation of [Ca^2+^]_cyto_ and reduction in [Ca^2+^]_m_. This was associated with increased miR-25 and miR-138 expression. Treatment with antagomiR against both miR-25 and miR-138 can restore MCU expression levels and reverse the pathophysiology of PAH (Hong et al., 2017).

**Figure 1:**
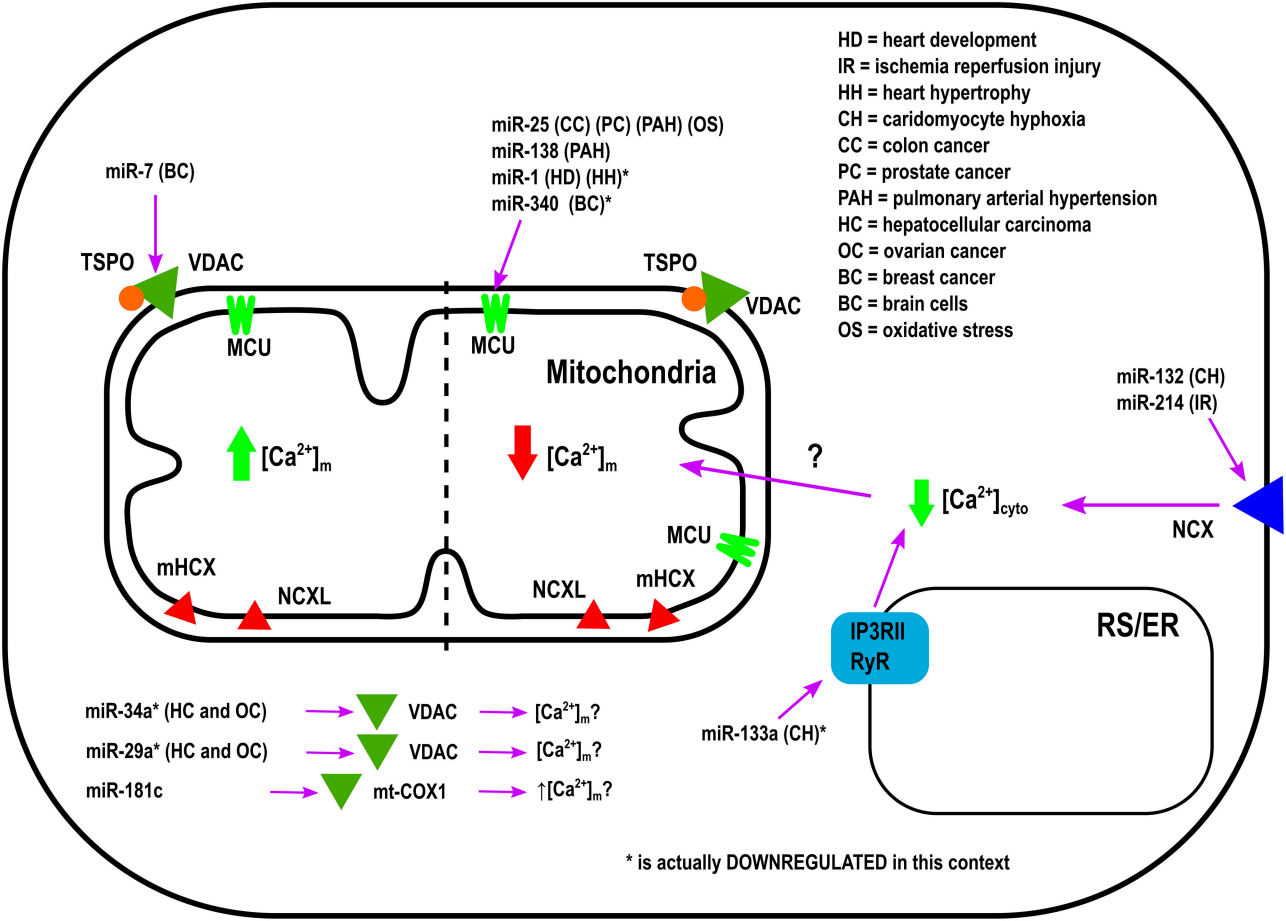
Mechanism of Action by which miRNAs regulate Ca^2+^-intake into the Mitochondria.

Overexpression of miR-25 in the heart protects myocytes from oxidative damage by reducing [Ca^2+^]_m_ levels through downregulation of MCU (Pan et al., 2015). On the other hand, it has been observed that miR-181c can directly target the mitochondrial encoded-gene, mt-COX1, which leads to increased ΔΨ_m_, which could be related to increased [Ca^2+^]_m_ influx and ROS production (Das et al., 2014). Zaglia et al., demonstrated a role for muscle-specific miRNAs (myomiR), such as miR-1 and miR-206, in heart tissue. This group successfully showed that miR-1 can directly regulate MCU expression during development in both mouse and human cardiac myocytes (Zaglia et al., 2017). During the neonatal phase, i.e., the first few days after birth, mitochondria are disorganized inside cells. Therefore, in this state the distance between mitochondria and ER or SR is far apart, and any Ca^2+^ release from the ER and/or SR could not influence [Ca^2+^]_m_ influx easily. However, by 7–14 days after birth, the membrane system matures, putting the ER, and/or SR closer to the mitochondrial membrane (Griffiths et al., 2010). In this phase, mitochondria are now exposed to ER and/SR high Ca^2+^ microdomains, i.e., Ca^2+^ release from the ER and/or SR influences [Ca^2+^]_m_ influx rapidly. Based on the intracellular location of mitochondria during the neonatal phase, miR-1 upregulation could protect mitochondria from [Ca^2+^]_m_ overload. This is due to the fact that miR-1 directly targets the mRNA of MCU (Zaglia et al., 2017). In contrast, during physiological and pathological hypertrophy where the ATP consumption rate is higher, downregulation of miR-1, and increased MCU levels were observed (Zaglia et al., 2017). Figure 1 summarizes the role of various miRNAs in the in-take of mitochondrial Ca^2+^.

## Other MicroRNAs involved in calcium regulation

Other miRNAs have been shown to indirectly regulate [Ca^2+^]_m_ overload and/or apoptosis. For example, during hypoxic conditions downregulation of miR-132 leads to increased Na^+^-Ca^2+^ exchanger 1 (NCX1) expression. Using rat cardiomyocytes it has been documented that miR-132 could influence [Ca^2+^]_m_ overload by binding to the 3′-UTR of NCX1 mRNA and modulating [Ca^2+^]_cyto_ (Hong et al., 2015). To release the intracellular H^+^ accumulation Na^+^-H^+^ exchanger (NHE) gets activated. Activated NHE release the H^+^ from the cell at the cost of Na^+^ overload. NCX1 plays an important role in releasing the Na^+^ from the cells to alleviate the intracellular Na^+^-overload. In cardiac hypertrophy, downregulation of miR-133a has been observed. This leads to higher IP3RII expression and increased [Ca^2+^]_cyto_, resulting in left ventricular remodeling and contractile dysfunction (Drawnel et al., 2012). On the contrary, miR-214, which increases its expression during ischemia/reperfusion (I/R) insult, has been shown to protect the heart from Ca^2+^ overload, heart failure, and apoptosis through targeting NCX1 (Aurora et al., 2012). Multiple pathophysiological conditions, such as hypertrophy and I/R, upregulate NCX1 in the heart (Sipido et al., 2002). Therefore, down-regulation of NCX1 can potentially be a therapeutic intervention from these pathophysiological conditions by reducing [Ca^2+^]_cyto_ overload and cell death. As it has been shown that miR-214 can directly bind to NCX1 mRNA, increased miR-214 expression can protect the heart from MI and I/R-stress (Aurora et al., 2012). Indeed, it has been observed that genetic deletion of miR-214 in mice results in impaired cardiac function and susceptibility to I/R injury (Aurora et al., 2012). After I/R, miR-214 significantly reduces [Ca^2+^]_cyto_ overload and protects cardiomyocytes from increased oxidative stress and cell death by reducing NCX1 activity. All of these effects are directly related to [Ca^2+^]_m_ (Aurora et al., 2012). Additionally, overexpression of miR-145 in rat ventricular cardiomyocytes reduces Ca^2+^/Calmodulin-Dependent Protein Kinase II (CaMKII) expression (together with other Ca^2+^ handling proteins), protecting myocytes from apoptosis induced by H_2_O_2_ (Cha et al., 2013).

As illustrated in Table 1, the miRNAs which are not directly involved in [Ca^2+^]_m_ handling, but they play an important role in buffering [Ca^2+^]_cyto_ (Shoshan-Barmatz et al., 2018). It has been shown that the alterations in [Ca^2+^]_cyto_ could influence [Ca^2+^]_m_ influx, either directly by influencing MCU, or indirectly via other MCU regulatory proteins, such as MICU1. Therefore, one miRNA can influence [Ca^2+^]_m_ entry by forming the RISC inside the mitochondria or at the cytoplasm. If the RISC is inside the mitochondrial compartment, then that miRNA will be termed as a “MitomiR.”

**Table 1:** List of miRNAs which regulate [Ca^2+^]m.

**miRNA and regulation**	**Δexpression**	**Type of cell/tissue**	**Physiological state/Model of study**	**Target**	**Origin**	**Consequence on mitochondrial calcium**	**Effect on Ca2+ regulated mechanisms**	**References**
miR-34a and miR-29a	Upregulation and dowregulation	HEK293T cells	Culture cells	VDAC1	Nuclear encoded	Not measured	Increased apoptosis	Tomasello et al., 2009; Bargaje et al., 2012
miR-7		SH-SY5Y neuroblastoma cells; mouse primary cortical neurons	Culture cells	VDAC1	Nuclear encoded	Reduce ${\text{Ca}}_{\text{m}}^{2+}$ efflux, ↑[Ca^2+^]_m_	Reduced ROS and apoptosis	Chaudhuri et al., 2016
miR-25	Overexpression	HeLa, colon cancer cell lines (HCT116, RKO, SW80 and WiDr cell lines), prostate cancer cell lines (PC3, LnCap and 22Rv1 cell lines)	Culture cells	MCU	Nuclear encoded	↓[Ca^2+^]_m_	Apoptosis resistance	Marchi et al., 2013
miR-340	Downregulation	Metastatic breast cancer cell lines (BT-474, MCF7, ZR-75-30, and MDA-MB-231)	Breast Cancer	MCU	Nuclear encoded	↑[Ca^2+^]_m_	Enhances the metastatic capacity of breast cancer cells by inducing a shift from oxidative to glycolytic metabolism	Yu et al., 2017
miR-25	Upregulation	PASMC	PAH	MCU	Nuclear encoded	↓[Ca^2+^]_m_	Apoptosis resistance	Hong et al., 2017
miR-138	Upregulation	PASMC	PAH	MCU	Nuclear encoded	↓[Ca^2+^]_m_	Apoptosis resistance	Hong et al., 2017
miR-25	Upregulation	H9C2 cell (embryonic rat ventricular myocyte line)	Oxidative stress by H_2_O_2_ treatment	MCU	Nuclear encoded	↓[Ca^2+^]_m_		Pan et al., 2015
miR-181c	Overexpression	Cardiomyocyte	Heart Failure	mt-COX1	Mitochondrial encoded (MitomiR)	↑[Ca^2+^]_m_?		Das et al., 2014
miR-1	Upregulation	Cardiac myocyte (human and mouse)	Postnatal cardiac growth	MCU	Nuclear encoded	↓${\text{Ca}}_{\text{m}}^{2+}$ uptake	Protection of mitochondria from high Ca2+ microdomains	Zaglia et al., 2017
miR-1	Downregulation	Mouse heart	Physiologycal and pathological hypertrophy	MCU	Nuclear encoded	↑${\text{Ca}}_{\text{m}}^{2+}$ uptake	Increase mitochondrial ATP production	Zaglia et al., 2017
miR-1	Downregulation	Human heart biopsies	myocardial hypertrophy due to aortic stenosis	MCU	Nuclear encoded	↑${\text{Ca}}_{\text{m}}^{2+}$ uptake	Increase mitochondrial ATP production	Zaglia et al., 2017
miR-132	Overexpression	NRCMs	Hypoxia	NCX1	Nuclear encoded	↑[Ca^2+^]_cyto_ → ↑[Ca^2+^]_m_?	Increased apoptosis	Hong et al., 2015
miR-133a	Dowregulation	AB in rats	Cardiac Hypertrophy	IP3RII	Nuclear encoded	↑[Ca^2+^]_cyto_ → ↑[Ca^2+^]_m_?	hypertrophic cardiomyocyte remodeling	Drawnel et al., 2012
miR-133a	Overexpression	HEK293, NRCMs, ARCMs	Culture cells	IP3RII	Nuclear encoded	↓[Ca^2+^]_cyto_ → ↓[Ca^2+^]_m_?	hypertrophic cardiomyocyte remodeling	Drawnel et al., 2012
miR-214	Dowregulation	miR-214 KO mice with LAD ligation (myocardial injury)	IR injuy	NCX1	Nuclear encoded	↑[Ca^2+^]_cyto_ → ↑[Ca^2+^]_m_?	Cells more sensitive to Ca2+ overload, increased apoptosis	Aurora et al., 2012
mir-145	Overexpression	Neonatal rat cardiomyocytes	Oxidative stress by H_2_O_2_ treatment	CaMKIIδ	Nuclear encoded	↓[Ca^2+^]_cyto_ → ↓[Ca^2+^]_m_?	ameliorates apoptosis	Cha et al., 2013

*Pulmonary arterial hypertension (PAH), pulmonary artery smooth muscle cell (PASMC), Ischemia reperfusion (IR), NADPH oxidases (NOXs), Neonatal rat cardiomyocytes (NRCMs), adult rat cardiomyocytes (ARCMs), aortic binding (AB), left anterior descending coronary artery (LAD)*.

## Concluding remarks

[Ca^2+^]_m_ is regulated by its influx and efflux through VDAC, the MCU complex, NCXL, and NHX. Therefore, the regulation of these Ca^2+^-sensing proteins can determine the fate of cellular activity. Recent studies have highlighted the importance of MitomiR on [Ca^2+^]_m_ handling and mitochondrial function, making these MitomiRs attractive as therapeutic targets. While there is still much work to be done with regards to miRNA import into the mitochondria, several groups have documented that MitomiRs can regulate [Ca^2+^]_m_ entry. The potential for miRNA therapeutics is limitless, and could be applied to any disease, including Alzheimers, Parkinson's, cancer, and heart failure, which are characterized by alterations in their MitomiR profiles. Indeed, it is true that when researchers across the world tried to regulate [Ca^2+^]_m_ by knocking down MCU protein in a mouse model, there were very conflicting results. In some studies, cardio-specific MCU knock-out proved detrimental against various stresses, such as ischemia/reperfusion injury. On the other hand, global MCU knock-out mice showed a minimal functional effect. MCU knock-out mice are slightly smaller compared to their wild type littermates and the mice have modest defects in skeletal muscle strength and some alterations in metabolic functions. Apart from these minor phenotypical defects, the global MCU knock-out mice are healthy. Therefore, alteration of MCU to regulate [Ca^2+^]_m_ will require extensive studies to fully uncover the underlying molecular mechanism(s) before proposing that a miRNA for regulating the MCU gene could be an effective strategy to achieve a therapeutic goal. However, the gate-keeper of MCU, MICU1, when altered showed very consistent outcomes in multiple groups. It is known that knocking down MICU1 is detrimental when [Ca^2+^]_cyto_ is low, whereas knocking-down MICU1 is beneficial when [Ca^2+^]_cyto_ is high. Studies have clearly shown that it is the ratio between MICU1 and MICU2 that plays a pivotal role in [Ca^2+^]_m_ influx. Therefore, it is fair to predict that miRNA(s) which can effectively bind to MICU1 and/or MICU2 mRNAs can influence the [Ca^2+^]_m._ In particular, there is great potential for treatment of conditions such as diabetes and cardiovascular disease with MitomiR(s) manipulation due to their impact on the mitochondria and overall cardiac function.

## Author contributions

CJDG and BR have performed the literature search. CJDG, BR, and SD wrote the manuscript. All the authors have read and consented to submit this manuscript.

### Conflict of interest statement

The authors declare that the research was conducted in the absence of any commercial or financial relationships that could be construed as a potential conflict of interest.

## Abbreviations

miRNA: microRNA
ROS: Reactive Oxygen Species
[Ca^2+^]_m_: Mitochondrial matrix calcium
mt-COX1: Cytochrome C Oxidase subunit 1
COX IV: Mitochondrial respiratory Complex IV
MCU: Mitochondrial Calcium Uniporter
PDH: Pyruvate Dehydrogenase
RISC: RNA-induced silencing complex
NCX1: Na^+^-Ca^2+^ exchanger 1.
